# Long working hours as a risk factor for atrial fibrillation: a multi-cohort study

**DOI:** 10.1093/eurheartj/ehx324

**Published:** 2017-07-13

**Authors:** Mika Kivimäki, Solja T. Nyberg, G. David Batty, Ichiro Kawachi, Markus Jokela, Lars Alfredsson, Jakob B. Bjorner, Marianne Borritz, Hermann Burr, Nico Dragano, Eleonor I. Fransson, Katriina Heikkilä, Anders Knutsson, Markku Koskenvuo, Meena Kumari, Ida E.H. Madsen, Martin L. Nielsen, Maria Nordin, Tuula Oksanen, Jan H. Pejtersen, Jaana Pentti, Reiner Rugulies, Paula Salo, Martin J. Shipley, Sakari Suominen, Töres Theorell, Jussi Vahtera, Peter Westerholm, Hugo Westerlund, Andrew Steptoe, Archana Singh-Manoux, Mark Hamer, Jane E. Ferrie, Marianna Virtanen, Adam G. Tabak, for the IPD-Work consortium

**Affiliations:** 1 Department of Epidemiology and Public Health, University College London, WC1E 6BT London, UK; 2 Clinicum, Faculty of Medicine, University of Helsinki, Tukholmankatu 8 B, 00290 Helsinki, Finland; 3 Finnish Institute of Occupational Health, Topeliuksenkatu 41 B, 00250 Helsinki, Finland; 4 Centre for Cognitive Ageing and Cognitive Epidemiology, University of Edinburgh, 7 George Square, EH8 9JZ, Edinburgh, UK; 5 Department of Social & Behavioral Sciences, Harvard T.H. Chan School of Public Health, 677 Huntington Avenue, Kresge Building 7th Floor, Boston, Massachusetts 02115, USA; 6 Department of Psychology and Logopedics, Faculty of Medicine, University of Helsinki, Haartmaninkatu 3, 00014 Helsinki, Finland; 7 Centre for Occupational and Environmental Medicine, Stockholm County Council, Solnavägen 4, 113 65 Stockholm, Sweden; 8 Institute of Environmental Medicine, Nobels väg 13, Karolinska Institutet, 171 77 Stockholm, Sweden; 9 National Research Centre for the Working Environment, Lersø Parkallé 105, 2100 Copenhagen ø, Denmark; 10 Bispebjerg University Hospital Copenhagen, Department of Occupational and Environmental Medicine, Bispebjerg Bakke 23_20F, DK-2400 Copenhagen NV, Denmark; 11 Federal Institute for Occupational Safety and Health (BAuA), Nöldnerstraße 40/42, 10317 Berlin, Germany; 12 Institute of Medical Sociology, Medical Faculty, University of Düsseldorf, Universitätsstraße 1, D-40225 Düsseldorf, Germany; 13 School of Health and Welfare, Jönköping University, Barnarpsgatan 39, 551 11 Jönköping, Sweden; 14 Stress Research Institute, Stockholm University, Frescati Hagväg 16 A, 114 19 Stockholm, Sweden; 15 Department of Health Services Research and Policy, London School of Hygiene and Tropical Medicine, UK 15-17 Tavistock Place, WC1H 9SH London, UK; 16 Clinical Effectiveness Unit, The Royal College of Surgeons, 35-43 Lincoln's Inn Fields, WC2A 3PE London, UK; 17 Department of Health Sciences, Mid Sweden University, Holmgatan 10, 851 70 Sundsvall, Sweden; 18 Institute for Social and Economic Research, University of Essex, Wivenhoe Park, Colchester, CO4 3SQ, Essex, UK; 19 AS3 Employment, AS3 Companies, Hasselager Centervej 35, DK-8260 VIBY J, Denmark; 20 Department of Psychology, Umeå University, SE-901 87 Umeå, Sweden; 21 Danish National Centre for Social Research, Herluf Trolles Gade 11, 1052 Copenhagen K, Denmark; 22 Department of Public Health and Department of Psychology, University of Copenhagen, Nørregade 10, PO Box 2177, 1017 Copenhagen K, Denmark; 23 Department of Psychology, University of Turku, Assistentinkatu 7, 20014 Turku, Finland; 24 University of Skövde, Högskolevägen 28, 541 45 Skövde, Sweden; 25 Department of Public Health, University of Turku, Joukahaisenkatu 3-5 A, 20520 Turku, Finland; 26 Turku University Hospital, Kiinamyllynkatu 4-8, 20521 Turku, Finland; 27 Department of Medical Sciences, Uppsala University, Akademiska sjukhuset, 75185 Uppsala, Sweden; 28 Inserm U1018, Centre for Research in Epidemiology and Population Health, Hôpital Paul-Brousse 16 avenue Paul Vaillant-Couturier, Bâtiment 15/16, 94807 Villejuif Cedex, France; 29 School of Sport, Exercise and Health Sciences, National Centre Sport & Exercise Medicine, Loughborough University, Epinal Way, Loughborough LE11 3TU, UK; 30 School of Social and Community Medicine, University of Bristol, Oakfield House, Oakfield Grove, Bristol BS8 2BN, UK; 31 1st Department of Medicine, Semmelweis University Faculty of Medicine, Budapest, Üllöi út 26, 1085 Budapest, Hungary

**Keywords:** Atrial fibrillation, Life stress, Risk factors, Cohort study

## Abstract

**Aims:**

Studies suggest that people who work long hours are at increased risk of stroke, but the association of long working hours with atrial fibrillation, the most common cardiac arrhythmia and a risk factor for stroke, is unknown. We examined the risk of atrial fibrillation in individuals working long hours (≥55 per week) and those working standard 35–40 h/week.

**Methods and results:**

In this prospective multi-cohort study from the Individual-Participant-Data Meta-analysis in Working Populations (IPD-Work) Consortium, the study population was 85 494 working men and women (mean age 43.4 years) with no recorded atrial fibrillation. Working hours were assessed at study baseline (1991–2004). Mean follow-up for incident atrial fibrillation was 10 years and cases were defined using data on electrocardiograms, hospital records, drug reimbursement registers, and death certificates. We identified 1061 new cases of atrial fibrillation (10-year cumulative incidence 12.4 per 1000). After adjustment for age, sex and socioeconomic status, individuals working long hours had a 1.4-fold increased risk of atrial fibrillation compared with those working standard hours (hazard ratio = 1.42, 95% CI = 1.13–1.80, *P* = 0.003). There was no significant heterogeneity between the cohort-specific effect estimates (*I*^2^ = 0%, *P* = 0.66) and the finding remained after excluding participants with coronary heart disease or stroke at baseline or during the follow-up (*N* = 2006, hazard ratio = 1.36, 95% CI = 1.05–1.76, *P* = 0.0180). Adjustment for potential confounding factors, such as obesity, risky alcohol use and high blood pressure, had little impact on this association.

**Conclusion:**

Individuals who worked long hours were more likely to develop atrial fibrillation than those working standard hours.

## Background

Atrial fibrillation is the most common cardiac arrhythmia and contributes to the development of several adverse health outcomes, such as stroke, heart failure, and multi-infarct dementia.^1–3^ Cardiovascular and respiratory disease, hypertension and left ventricular hypertrophy are risk factors for atrial fibrillation.^2^^,^^4^^,^^5^ Additionally, findings from observational studies have been used to suggest that maintaining a lifestyle that reduces the risk of cardiovascular disease—avoidance of obesity, smoking and heavy alcohol consumption—may also have a positive impact on rates of atrial fibrillation.^6–8^

Although the 2016 European Guidelines for cardiovascular disease prevention acknowledges psychosocial stress at work as a potential risk factor for cardiovascular disease,^9^ citing evidence that show long working hours to be associated with increased stroke risk,^10^ little is known about the role of long working hours as a potential risk factor of atrial fibrillation. In principle, stress and long working hours may enhance functional re-entry, repetitive pulmonary vein and atrial firing,^11^^,^^12^ and autonomic nervous system abnormalities,^13^ inducing arrhythmia vulnerability.^14^ Thus, some studies have found that stress and ‘exhaustion’ predict symptomatic atrial fibrillation.^15^^,^^16^ However, this evidence is uncertain because it is based on small study samples. Accordingly, we conducted a large-scale study on long working hours and incident atrial fibrillation in the general population using data from cohort studies participating in the Individual-Participant-Data Meta-analysis in Working Populations (IPD-Work) Consortium.^10^^,^^17^^,^^18^

## Methods

### Participants

In ten cohort studies of the IPD-Work Consortium, data on working hours and atrial fibrillation were available, although in two studies (the Intervention Project on Absence and Well-being and the Work, Lipids and Fibrinogen study Norrland) the low number of participants with long working hours (*n* = 6 and 55, respectively) and atrial fibrillation during the follow-up (*n* = 0 among participants working long hours in both studies) precluded inclusion of these studies in the analysis. The remaining eight studies were included in the analyses: the Copenhagen Psychosocial Questionnaire Study (COPSOQ) I and COPSOQ-II, the Danish Work Environment Cohort Study (DWECS), the Finnish Public Sector Study (FPS), the Health and Social Support study (HeSSup), the PUMA study, the Whitehall II study, and the Work, Lipids and Fibrinogen study (WOLF), Stockholm (see Supplementary material online, *Appendix S1*). Most of these were multi-purpose studies designed to examine health effects across a range of risk factors, including those related to workplace. The analytic sample comprised 85 494 participants (29 579 men and 55 915 women) from the UK, Denmark, Sweden, and Finland who were free of atrial fibrillation at baseline (1991–2004). All studies were approved by the relevant local or national ethics committee and all participants gave informed consent to participate.

### Assessment of working hours and covariates at baseline

Working hours were assessed at baseline which was between 1991 and 2004 depending on the cohort study. As in previous studies, we classified working hours into categories of ‘less than 35 h’, ‘35–40 h’, ‘41–48 h’, ‘49–54 h’, and ‘≥55 h/week’.^10^^,^^17^^,^^18^ The first category includes part-time workers and the second category is the reference group of full-time workers with standard working hours. The category of 41–48 h/week includes those working more than standard hours but still in accordance with the European Union Working Time Directive (2003/88/EC), which guarantees employees the right to limit weekly working time at 48 h on average. The remaining two categories include working times beyond this threshold, with the top category of 55 or more hours per week being the most commonly used definition for long working hours in medical research.^10^^,^^17–20^

Pre-defined, harmonized covariates included potential confounders, such as age, sex, and socioeconomic status (SES; high, intermediate, low, unknown), and potential mediators, such as smoking (current, ex, never smoker), body mass index (BMI, calculated as weight (in kilograms)/height (in meters) squared and categorized according to the WHO classification: <18.5, 18.5–24.9, 25.0–29.9, 30.0–34.9, ≥35 kg/m^2^), physical activity (sedentary, moderately active, highly active), and alcohol consumption (non-use; moderate, women: 1–14 drinks/week, men: 1–21 drinks/week; intermediate, women: 15–20 drinks/week; men: 22–27 drinks/week; risky: women: 21 or more drinks/week, men: 28 or more drinks/week).

As ascertainment of atrial fibrillation in the Whitehall II study was by electrocardiogram (ECG), the gold standard method, and the study included the widest range of atrial fibrillation risk factors of all IPD-Work studies, a further set of analyses were undertaken in those data only. Additional non-cardiovascular and cardiovascular risk factors at baseline deemed to act as potential confounders or mediators of the long working hours-atrial fibrillation relationship included:^5^^,^^21^ Prevalent infection/high systemic inflammation defined using serum C-reactive protein (high-sensitivity immunonephelometric assay in a BN ProSpec nephelometer; values >10 mg/L); self-reported respiratory illness and doctor-diagnosed heart trouble (including valve disease and congestive heart failure); left ventricular hypertrophy (Minnesota codes 3-1, 3-3, 3-4); diabetes mellitus (defined as fasting glucose >7.0 mmol/L or a 2-h post load glucose >11.1 mmol/L during a 75 g oral glucose tolerance test, or self-reported doctor-diagnosed diabetes); depressive and anxiety symptoms using the General Health Questionnaire caseness;^22^ systolic blood pressure (the average of two readings taken in the sitting position after 5 min of rest with the Hawksley random-0 sphygmomanometer); use of antihypertensive medication; total and high-density-lipoprotein (HDL) cholesterol concentrations (measured by automated enzymatic colorimetric methods).

To examine whether cardiovascular disease preceded or followed atrial fibrillation, we assessed coronary heart disease and stroke events at baseline and follow-up. Coronary heart disease was denoted by diagnostic codes I21–I22 in ICD-10, 410 in ICD-9 (in hospitalization data) or using the MONICA criteria (Whitehall II study clinical examination).^23^ Coronary death included diagnostic codes I20–I25 in ICD-10, 410–414 in ICD-9. Stroke included diagnostic codes I60, I61, I63, I64 in ICD-10 and 430, 431, 433, 434, 436 in ICD-9.

### Outcome ascertainment

In WOLF, HeSSup, PUMA, FPS, DWECS, COPSOQ-I, and COPSOQ-II, cases of atrial fibrillation at baseline and follow-up were identified using electronic patient records of hospitalizations and deaths [International Statistical Classification of Diseases and Related Health Problems (ICD) diagnostic codes I48 (ICD-10), 427.3 (ICD-9) or 427.4 (ICD-8)]. In FPS and HeSSup, atrial fibrillation cases were additionally identified from the nationwide drug reimbursement register for the treatment of this condition. In that register, entitlement to reimbursement is based on a detailed medical examination and predefined criteria for the diagnosis. In the Whitehall II study, atrial fibrillation was assessed using resting ECGs (Minnesota code 83x) at baseline in 1991 and at follow-up examinations in 1997, 2003, and 2008. In each study, participants with any indication of pre-existing atrial fibrillation in electronic health records or ECG at baseline were excluded (*n* = 250).

### Statistical analysis

We analysed anonymized or pseudonymized individual-level data from each cohort. We studied the associations between long working hours and baseline covariates using logistic regression for dichotomous covariates (obesity, physical inactivity, current smoking, risky alcohol use, infection/high systemic inflammation, respiratory disease, heart trouble, left ventricular hypertrophy, diabetes, depressive and anxiety symptoms, antihypertensive medication) and analysis of variance for continuous covariates (systolic blood pressure, total, and HDL cholesterol) with adjustment for age (continuous variable), sex and SES (categorical variables). To examine the extent to which incident atrial fibrillation was due to pre-existing cardiovascular disease, we computed the proportion of incident atrial fibrillation cases who had a record of cardiovascular disease (coronary heart disease or stroke) before atrial fibrillation was first recorded.

After confirming that the proportional hazards assumptions were not violated, we used Cox proportional hazards models to generate hazard ratios and 95% confidence intervals (CI) for long working hours (55 h or more per week) compared with standard (35–40) working hours (reference) in predicting incident atrial fibrillation in participants free of this arrhythmia at baseline. In the basic statistical model, effect estimates were adjusted for age (continuous variable), sex, and SES (categorical variable) at baseline. Adjustment for SES is important because long working hours were more common in participants with high SES (6.9% worked long hours) relative to those in low SES group (4.6%). To examine whether the association between long working hours and atrial fibrillation was mediated by poor lifestyle factors, adjustments were made for smoking (never, ex-, current smoker), alcohol consumption (non-use, moderate, risky), BMI (categorical), and physical activity (inactive, moderately active, highly active) at baseline. In analyses carried out in the Whitehall II study, additional adjustments were made for doctor-diagnosed heart abnormalities, infection/high systemic inflammation, respiratory disease, heart problems, left ventricular hypertrophy, diabetes mellitus, depressive and anxiety symptoms, use of antihypertensive medication (all dichotomous variables), systolic blood pressure and total and HDL-cholesterol (continuous variables), all measured at baseline.

Meta-analysis, based on random-effects modelling, was used to combine results from each cohort. We examined heterogeneity of the cohort-specific estimates using the *I*^2^ statistic (a higher value indicating a greater degree of heterogeneity). In sensitivity analyses, we examined the association separately in men and women, by age group (<50 vs. >50 years at baseline) and by socioeconomic status (high, intermediate, low). We also stratified the analysis by the method of case ascertainment to examine whether the association between long working hours and atrial fibrillation was attenuated when the ascertainment was based on electronic health records from registers of hospital admissions, deaths and drug reimbursement as compared with ECG assessment.

The statistical software SAS (version 9.4) was used to analyse study-specific data and Stata (MP version 13.1) was used to compute the meta-analyses.

## Results

Of the 85 494 participants, 35% were men and the mean age was 43.4 years (range 17–70) at baseline (*Table 1*). During the mean follow-up of 10.0 years, 1061 participants were diagnosed with atrial fibrillation (10-year cumulative incidence 12.4 per 1000). In 71.4% of cases, atrial fibrillation was diagnosed before the age of 65 (see Supplementary material online, *Appendix S2*). This is as expected given the young mean age and length of follow-up. Of the incident atrial fibrillation cases, 86.7% had no cardiovascular disease during the study period whereas 10.2% of incident cases of atrial fibrillation had pre-existing cardiovascular disease when atrial fibrillation was first recorded (see Supplementary material online, *Appendix S3*).

**Table 1:** Baseline characteristics of participants by atrial fibrillation status at follow-up

	All * N* = 85 494	Incident cases *N* = 1061	Non-cases * N* = 84 433
Age, years			
Mean	43.4	51.6	43.3
Range	(17–70)	(21–69)	(17–70)
Sex, *N* (*%*)			
Men	29 579 (34.5)	678 (63.9)	28 901 (34.2)
Women	55 915 (65.5)	383 (36.1)	55 532 (65.8)
Socioeconomic status, *N* (*%*)			
High	22 555 (26.4)	336 (31.7)	22 219 (26.3)
Intermediate	41 570 (48.6)	432 (40.7)	41 138 (48.7)
Low	19 625 (23.0)	279 (26.3)	19 346 (22.9)
Unknown	1744 (2.0)	14 (1.3)	1730 (2.0)
Country, *N* (*%*)			
UK	6649 (7.8)	224 (21.1)	6425 (7.6)
Denmark	12 563 (14.7)	161 (15.2)	12 402 (14.7)
Sweden	5551 (6.5)	131 (12.3)	5420 (6.4)
Finland	60 731 (71.0)	545 (51.4)	60 186 (71.3)

All participants were free of atrial fibrillation at study baseline.

A total of 4484 (5.2%) participants worked ≥55 h/week and 53 468 (62.5%) worked standard 35–40 hours at baseline. Long working hours were associated with a slightly poorer lifestyle profile at baseline characterized by a higher prevalence of obesity, leisure-time physical inactivity, smoking and risky alcohol use (*Table 2*, Supplementary material online, *Appendix S4*). Analysis of further baseline covariates in the Whitehall II study show that participants working long hours were more likely to have depressive and anxiety symptoms and less likely to have left ventricular hypertrophy than those working standard hours.

**Table 2:** Differences in lifestyle, biological and psychological factors between individuals working long (≥55 h/week) and standard (35–40 h/week) working hours

	Working hours category			
Baseline characteristic	Long	Standard		
IPD-Work cohorts^a^	Prevalence (%)		Odds ratio^b^ (95% CI)	*P*-value
Obese	11.8	10.5	1.34 (1.17 to 1.54)	<0.0001
Physically inactive	21.7	19.1	1.18 (1.07 to 1.30)	0.0007
Smoking	24.9	22.3	1.15 (1.02 to 1.31)	0.026
Risky alcohol use	8.4	5.7	1.18 (1.04 to 1.33)	0.0084
Whitehall II^c^				
Infection/high inflammation	2.1	2.2	1.26 (0.66 to 2.42)	0.48
Respiratory disease	7.9	6.5	1.30 (0.91 to 1.84)	0.15
Heart trouble (incl. valve disease)	7.4	7.9	0.88 (0.62 to 1.24)	0.45
Left ventricular hypertrophy	8.6	9.9	0.70 (0.50 to 0.96)	0.028
Diabetes mellitus	1.4	2.6	0.74 (0.35 to 1.57)	0.43
Depressive and anxiety symptoms	27.2	20.0	1.57 (1.27 to 1.95)	<0.0001
Antihypertensive medication	3.3	6.3	0.66 (0.40 to 1.08)	0.10
	Unadjusted mean		Mean difference^a^ (95% CI)	*P*-value
Systolic blood pressure (mmHg)	119.3	119.9	−1.1 (−2.3 to 0.1)	0.071
Total cholesterol (mmol/L)	6.4	6.4	0.0 (−0.1 to 0.1)	0.49
HDL-cholesterol (mmol/L)	1.4	1.4	−0.01 (−0.04 to 0.02)	0.57

a 4486 participants with long working hours and 53 502 participants with standard working hours.

b Odds ratios and mean differences for long compared with standard hours with risk factor as the outcome. Adjustment for age, sex and socioeconomic status.

c 584 participants with long working hours and 3016 participants with standard working hours.

**Figure 1:**
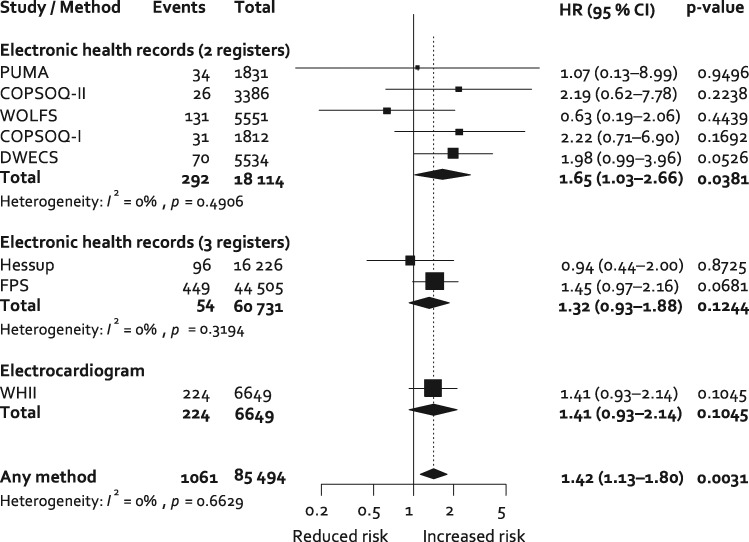
Random-effects meta-analysis of the association of long vs. standard working hours with incident atrial fibrillation adjusted for age, sex, and socioeconomic status. HR, hazard ratio.

In age, sex and SES-adjusted analyses, participants working long hours were at increased risk of incident atrial fibrillation: the hazard ratio compared with those working standard hours is 1.42 (95% CI 1.13–1.80, *P* = 0.0031) (*Figure 1*). There was little heterogeneity in the cohort-specific estimates: *I*^2^ = 0%, *P* = 0.66. Additional adjustment for lifestyle factors marginally attenuated the association between long vs. standard working hours and incident atrial fibrillation (1.41, 95% CI 1.10–1.80, *P* = 0.0059, *I*^2^ = 0%, *P* = 0.62) (see Supplementary material online, *Appendix S5*). The association between long working hours and atrial fibrillation remained after adjustment for pre-existing coronary heart disease at the time of atrial fibrillation diagnosis (1.41, 95% CI 1.12–1.78, *P* = 0.0039) and excluding participants with cardiovascular disease at baseline (*N* = 549, hazard ratio 1.41, 95% CI 1.11–1.79, *P* = 0.0054) or cardiovascular disease at baseline or follow-up (*N* = 2006, hazard ratio = 1.36, 95% CI = 1.05–1.76, *P* = 0.0180).

As the Whitehall II study had available data on several other potential risk factors for atrial fibrillation, further adjustments were performed using data from this cohort. The hazard ratio for long vs. standard working hours as a predictor of incident atrial fibrillation was 1.41 (95% CI 0.93–2.14, *P* = 0.1045, *N* = 6649, 224 incident cases of atrial fibrillation) after adjustment for age, sex, and SES; this is close to that observed in the total population (*Figure 1*). Additional adjustment for lifestyle factors, infection/high systemic inflammation, respiratory disease, doctor-diagnosed heart trouble (including valve disease and congestive heart failure), left ventricular hypertrophy, diabetes mellitus, depressive and anxiety symptoms, systolic blood pressure, antihypertensive medication, total and HDL-cholesterol had little effect on this estimate (1.42, 95% CI 0.91–2.23, *P* = 0.12, *N* = 5867, 195 incident cases of atrial fibrillation).

**Figure 2:**
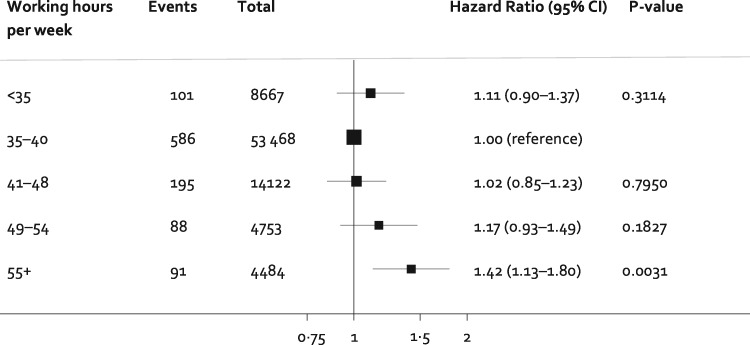
Association of categories of weekly working hours with incident atrial fibrillation. Estimates are adjusted for age, sex, and socioeconomic status.

*Figure 2* shows the shape of the association between all the categories of working hours and incident atrial fibrillation. There was a dose-response gradient with hazard ratios of 1.02, 1.17, and 1.42 for 41–48, 49–54 and ≥55 working hours per week compared with standard 35–40 working hours per week.

### Sensitivity analysis

In meta-analysis stratified by method of ascertainment of atrial fibrillation (*Figure 1*), the age-, sex- and SES-adjusted hazard ratio for long working hours compared with standard working hours was 1.41, 95% CI 0.93–2.14, *P* = 0.105, for the one study using electrocardiogram, 1.32, 95% CI 0.93–1.88, *P* = 0.124, for the two studies using records from hospital admissions, death and drug reimbursements, and 1.65, 95% CI 1.03–2.66, *P* = 0.038, for the five studies using records from hospital admissions and deaths only. In stratified analyses, the association between long working hours and atrial fibrillation did not differ between men and women (*P* = 0.267), participants younger than 50 and those 50 years or older at baseline (*P* = 0.704) or by socioeconomic group (*P* = 0.186).

## Discussion

It was found in this multi-cohort study of 85 494 men and women that those working 55 h or more a week had an approximately 40% higher risk of atrial fibrillation compared with those working a standard 35–40-h week. Nine out of ten incident atrial fibrillation cases occurred among those free of pre-existing or concurrent cardiovascular disease, suggesting that the observed excess risk of atrial fibrillation is likely to reflect the effect of long working hours rather than the effect of pre-existing or concurrent cardiovascular disease. Multivariable adjusted analyses showed that the association was not attributable to socioeconomic circumstances, lifestyles or common risk factors for atrial fibrillation. In combination, these findings suggest that long working hours is a risk factor for atrial fibrillation.

We are not aware of other studies on long working hours and atrial fibrillation, although our investigation is in agreement with small-scale studies linking other work-related stressors, such as job strain, to this condition.^24^^,^^25^ The mechanisms underlying the association between long working hours and atrial fibrillation are not known. A recent systematic review of observational evidence from over 20 million men and women found that obesity, smoking, hypertension, and high systemic inflammation were associated with an increased risk of atrial fibrillation, whereas evidence on cholesterol and physical activity was inconsistent.^21^ Other studies have also suggested that high alcohol consumption and obesity-related conditions, such as sleep apnea, may have a role in the aetiology of atrial fibrillation.^26^^,^^27^ In the present study, the prevalence of obesity, smoking, physical inactivity and high alcohol consumption was higher in individuals working long hours than in the standard working hours group, but the difference was small (<3 percentage points). Similarly, there appeared to be no difference in systemic inflammation, systolic blood pressure or cholesterol. As such, classic risk factors for atrial fibrillation are unlikely to mediate the association between long working hours and atrial fibrillation. In contrast, there has been the suggestion of a link between extensive overtime working and autonomic nervous system abnormalities,^13^ a risk factor for atrial fibrillation.^14^^,^^28^^,^^29^ As such, stress-related mechanisms that may trigger arrhythmia, such as autonomic dysfunction, might be a more promising focus for future studies on long working hours and atrial fibrillation than mediation via classic cardiovascular disease risk factors.

In absolute terms, the increased risk of atrial fibrillation among individuals with long working hours is relatively modest. The number of cases varied between 13 and 449 in the included studies; none of the study-specific associations between long working hours and atrial fibrillation reached statistical significance at conventional levels. In contrast, the association was highly significant in our pooled sample including a total of 1061 incident atrial fibrillation cases. The method of atrial fibrillation ascertainment was not uniform across studies—in only one study were participants repeatedly assessed using an electrocardiogram, the gold standard method, while in two other studies cases were identified via records from hospital admissions, death certificates or drug reimbursements, and in five studies only records from hospital admissions and deaths were available. The occurrence of atrial fibrillation is likely to be underestimated in the seven record linkage studies as they may miss undiagnosed and mildly symptomatic cases. While the study using an electrocardiogram is stronger methodologically, atrial fibrillation can be episodic and these “paroxysmal” cases are difficult to identify even with an electrocardiogram. Importantly, however, the relative risk of atrial fibrillation among individuals with long working hours was similar across the studies irrespective of the method of ascertainment: 1.4 in the study with ECG ascertainment, 1.3 in studies using hospital, prescription and death records, and 1.4 in those with hospital and death records only. This suggests that misclassification was random in terms of participants’ working hours and has therefore not caused a significant bias.

While novel and large in scale, our study has several limitations. First, as described, heterogeneous assessment of atrial fibrillation is a drawback. Second, working hours and lifestyle factors were only assessed at study induction. As working hours vary over time, our findings may under- or overestimate the true effect due to imprecise measurement of long-term exposure. Similarly, a lack of repeat measurement of lifestyle factors prevented us from examining potential behavioural mediators in the association between long working hours and atrial fibrillation. Third, the overall study population (*N* = 85 494) included more women (65%) than men (35%). This was because the largest cohort—the Finnish Public Sector study (*N* = 44 505)—is 81% female reflecting the sex distribution of public sector workers in Finland at the time of study enrolment. That there was no significant sex difference in the association between working hours and atrial fibrillation suggests our sex-adjusted analyses of men and women combined provide an accurate estimation of the association. Fourth, it is noteworthy that despite differences between the studies in terms of year of recruitment (range from 1991 to 2004), study population, location, methodology and settings, there was no significant heterogeneity in study-specific estimates of the association between long working hours and risk of atrial fibrillation. This supports the robustness of the main finding.

## Conclusion

Our findings raise the hypothesis that long working hours may affect the risk of atrial fibrillation (Summarizing Figure). We showed that employees working long hours were 40% more likely to develop this cardiac arrhythmia than those working standard hours. As this association appeared to be independent of known risk factors for atrial fibrillation, further research is needed to determine mechanisms underlying the link between long working hours and atrial fibrillation. Furthermore, the participants of this study were from the UK, Denmark, Sweden, and Finland. Although there is no reason to assume that the association would be dependent on geographical region, the generalizability of our findings to other countries remains to be confirmed.

**Summarizing Figure:**
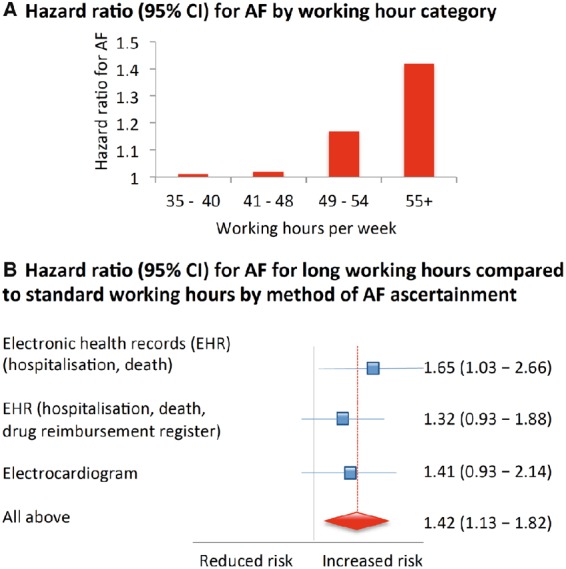
Association between working hours and risk of atrial fibrillation (AF) in 85 494 men and women free of AF at baseline. During the mean follow-up of 10.0 years, 1061 developed AF. The figure shows that persons who worked 55 hours or more per week had a 1.4-fold increased risk of AF compared to those working standard 35–40 weekly hours (*A*). This estimate did not vary according to the method of AF ascertainment (*B*).

## Supplementary material

Supplementary material is available at *European Heart Journal* online.

## Authors’ contributions

M.K. along with A.T. developed the hypothesis. S.N. and I.M. performed statistical analyses. M.K. wrote the first draft; all authors contributed to study concept and design, analysis and interpretation of data, and drafting or critical revision of the manuscript for important intellectual content, or, in addition, data acquisition.

## Funding

IPD-Work consortium was supported by NordForsk, a Nordic Research Programme on Health and Welfare, the EU New OSH ERA research programme, the Finnish Work Environment Fund, Finland, the Swedish Research Council for Working Life and Social Research, Sweden, Danish National Research Centre for the Working Environment, Denmark. NordForsk and the UK Medical Research Council (K013351 to M.K.).

**Conflict of interest**: none declared.

## Supplementary Material

Supplementary Appendix
