# Supplementary material for: Long working hours as a risk factor for atrial fibrillation: a multi-cohort study
Source: Eur Heart J. 2017 Jul 13;38(34):2621–8. doi: 10.1093/eurheartj/ehx324 (PMC5837794; doi:10.1093/eurheartj/ehx324)

Web appendix 1: Description of the cohort studies

Copenhagen Psychosocial Questionnaire version I (COPSOQ-I)[1]

The COPSOQ-I is a prospective cohort study of a random sample of Danish residents selected from the Danish population register. The participants were 20-60 years of age and in paid employment at study baseline in 1997. A baseline questionnaire and an invitation to take part was posted to 4 000 people and 2 454 individuals agreed to participate, of which 1858 were gainfully employed. In Denmark, questionnaire- and register-based studies do not require approval from the Danish National Committee on Biomedical Research Ethics (Den Centrale Videnskabetiske komité). COPSOQ-I was approved by and registered with the Danish Data protection agency (registration number: 2008 - 54 - 0553). Following the European Socio-economic Classification based on the ILO International Standard Classification of Occupations, three levels of SES were defined: high (e.g. lawyers, physicians), intermediate (e.g. office clerks, administrative associate professionals) and low (e.g. care workers, cleaners).

Copenhagen Psychosocial Questionnaire version II (COPSOQ-II)[2]

COPSOQ-II was carried out in 2004-2005. It included a follow up of respondents from COPSOQ I and also a representative sample of Danish residents aged 20-60 at study baseline. The questionnaire was sent to 8 000 individuals from the random sample and 4 732 individuals responded. The questionnaire could be completed using the postal questionnaire or via internet. COPSOQ-II was approved by and registered with the Danish Data protection agency (registration number: 2004-54-1493). Following the European Socio-economic Classification based on the ILO International Standard Classification of Occupations, three levels of SES were defined: high (e.g. lawyers, physicians), intermediate (e.g. office clerks, administrative associate professionals) and low (e.g. care workers, cleaners).

Danish Work Environment Cohort Study (DWECS)[3]

DWECS is a split panel survey of working age Danish people. The cohort was established in 1990, when a simple random sample of men and women, aged 18-59, was drawn from the Danish population register. The participants have been followed up at five year intervals and data from the year 2000 was used for IPD-Work. That year 11 437 individuals were invited to participate and 8 583 agreed to do so. In Denmark, questionnaire- and register-based studies do not require ethics committee approval. DWECS was approved by and registered with the Danish Data protection agency (registration number: 2007-54-0059). Following the European Socio-economic Classification based on the ILO International Standard Classification of Occupations, three levels of SES were defined: high (e.g. lawyers, physicians), intermediate (e.g. office clerks, administrative associate professionals) and low (e.g. care workers, cleaners).

Finnish Public Sector study (FPS)[4]

The Finnish Public Sector study is a prospective cohort study comprising the entire public sector personnel of 10 towns (municipalities) and 21 hospitals in the same geographical areas. Participants, who were recruited from employers' records in 2000-2002, were individuals who had been employed in the study organizations for at least six months prior to data collection. 48 592 individuals (9 337 men and 39 255 women aged 17 to 65) responded to the questionnaire. Ethical approval was obtained from the ethics committee of the Finnish Institute of Occupational Health. Participants' occupational titles from the employers' records (>1900 different five-digit occupational titles) were classified into high (upper non-manual including e.g. physicians, teachers); intermediate (lower non-manual, e.g. technicians, registered nurses); and low (manual, e.g. cleaners, maintenance workers) SES on the basis of Statistics Finland's occupational classification system.

Health and Social Support (HeSSup) [5]

The Health and Social Support (HeSSup) study is a prospective cohort study of a stratified random sample of the Finnish population in the following four age groups: 20–24, 30–34, 40–44, and 50–54. The participants were identified from the Finnish population register and posted an invitation to participate, along with a baseline questionnaire, in 1998. A total of 25 898 individuals responded to the questionnaire. The Turku University Central Hospital Ethics Committee approved the study. SES was defined based on participant's highest educational achievement as high (higher education, university) intermediate (higher or lower secondary, eg college/vocational school), and low (primary education only).

Burnout, Motivation and Job Satisfaction study (Danish acronym: PUMA) [6]

Burnout, Motivation and Job Satisfaction study (Danish acronym: PUMA) is an intervention study of burn-out among employees in the human service sector. Selection criteria for the participating organizations was that they had between 200 and 500 employees, that occupational groups within each organization were willing to participate and that the organizations would commit to the entire five-year study period. Participants gave consent to having their national identity numbers collected and used in later record linkages to Danish hospitalization and cause of death registries (Hospitalsindlæggelsesregisteret, Dødsårsagsregisteret). At study baseline in 1999-2000, 1 914 participants agreed to take part. PUMA was approved by the Scientific Ethical Committees (Videnskabsetisk Komiteer) in the counties in which the study was conducted and approved by and registered with the Danish Data Protection Agency (registration number: 2000-54-0048). Following the European Socio-economic Classification based on the ILO International Standard Classification of Occupations, three levels of SES were defined: high (e.g. lawyers, physicians), intermediate (e.g. office clerks, administrative associate professionals) and low (e.g. care workers, cleaners).

Whitehall II [7]

The Whitehall II study is a prospective cohort study set up to investigate socioeconomic determinants of health. At study baseline in 1985-1988, 10 308 civil service employees (6 895 men and 3 413 women) aged 35-55 and working in 20 civil service departments in London were invited to participate in the study. The Whitehall II study protocol was approved by the University College London Medical School committee on the ethics of human research. Written informed consent was obtained at each data collection wave. SES was assessed using civil service occupation-based employment grade categories: high (Administrative grades 1 to 7), intermediate (Professional and Executive grades including senior executive officers, higher executive officers and executive officers) and low (clerical grades including office support staff).

WOLF (Work, Lipids, and Fibrinogen) Stockholm [8]

The WOLF (Work, Lipids, and Fibrinogen) Stockholm study is a prospective cohort study of 5499 people with data on working conditions, aged 19–70 and working in companies in Stockholm county. At study baseline the participants underwent a clinical examination and completed a set of health questionnaires. For WOLF Stockholm, the baseline assessment was undertaken at 20 occupational health units between November 1992 and June 1995. The Regional Research Ethics Board in Stockholm, and the ethics committee at Karolinska Institutet, Stockholm, Sweden approved the study. The 3 levels of SES were high (professional, higher manager), intermediate (technical, lower management, non-manual), low (skilled and unskilled manual).

References

1. Kristensen TS, Hannerz H, Hogh A, Borg V. The Copenhagen Psychosocial Questionnaire--a tool for the assessment and improvement of the psychosocial work environment. *Scand J Work Environ Health.* 2005; 31(6): 438-49.

2. Pejtersen JH, Kristensen TS, Borg V, Bjorner JB. The second version of the Copenhagen Psychosocial Questionnaire. *Scand J Public Health.* 2010; 38(3 Suppl): 8-24.

3. Feveile H, Olsen O, Burr H, Bach E. Danish Work Environment Cohort Study 2005: From idea to sampling design. *Statistics in Transition.* 2007; 8(3): 441-58.

4. Kivimäki M, Lawlor DA, Smith GD, et al. Socioeconomic Position, Co-Occurrence of Behavior-Related Risk Factors, and Coronary Heart Disease: the Finnish Public Sector Study. *Am J Public Health.* 2007; 97(5): 874-9.

5. Korkeila K, Suominen S, Ahvenainen J, et al. Non-response and related factors in a nation-wide health survey. *Eur J Epidemiol.* 2001; 17(11): 991-9.

6. Borritz M, Rugulies R, Bjorner JB, Villadsen E, Mikkelsen OA, Kristensen TS. Burnout among employees in human service work: design and baseline findings of the PUMA study. *Scand J Public Health.* 2006; 34(1): 49-58.

7. Marmot MG, Smith GD, Stansfeld S, et al. Health inequalities among British civil servants: the Whitehall II study. *Lancet.* 1991; 337(8754): 1387-93.

8. Peter R, Alfredsson L, Hammar N, Siegrist J, Theorell T, P. W. High effort, low reward, and cardiovascular risk factors in employed Swedish men and women: baseline results from the WOLF Study. *J Epidemiol Community Health.* 1998; 52: 540-7.

Web appendix 2: Incident atrial fibrillation by age

eFigure 1. Cumulative number of incident atrial fibrillation cases by age (N=1061 cases in total)

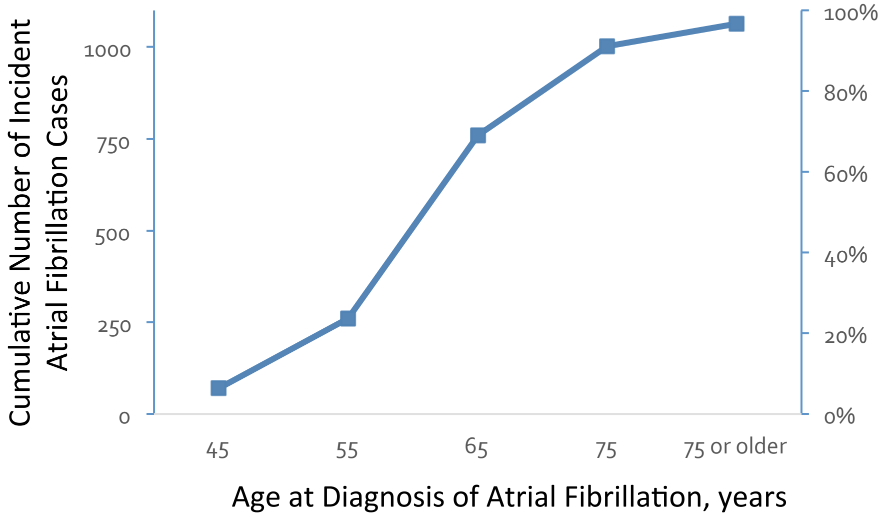

Web appendix 3: Coronary heart disease and stroke in employees with incident atrial fibrillation by working hours and time of onset

eTable 1. Cardiovascular disease (coronary heart disease or stroke) in employees with incident atrial fibrillation by working hours and time of onset

	Incident atrial fibrillation cases		
	**All**	**Long hours** (≥55 hours per week)	**Standard hours** (35-40 hours per week)
Atrial fibrillation with no pre-existing or comorbid cardiovascular disease at baseline or follow-up	790 (86.7%)	58 (84.1%)	448 (87.2%)
Cardiovascular disease diagnosed before or at the same time as atrial fibrillation	93 (10.2%)	9 (13.0%)	50 (9.7%)
Cardiovascular disease diagnosed after atrial fibrillation	28 (3.1%)	2 (2.9%)	16 (3.1%)
Total*	911 (100%)	69 (100%)	514 (100%)

*150 (14%) atrial fibrillation cases were not be included in this analysis because atrial fibrillation occurred after the end of follow-up for cardiovascular disease

Web appendix 4: Random-effects meta-analyses of long working hours and lifestyle-related factors

eFigure 2. Age, sex and socioeconomic status adjusted association between long working hours and obesity at baseline

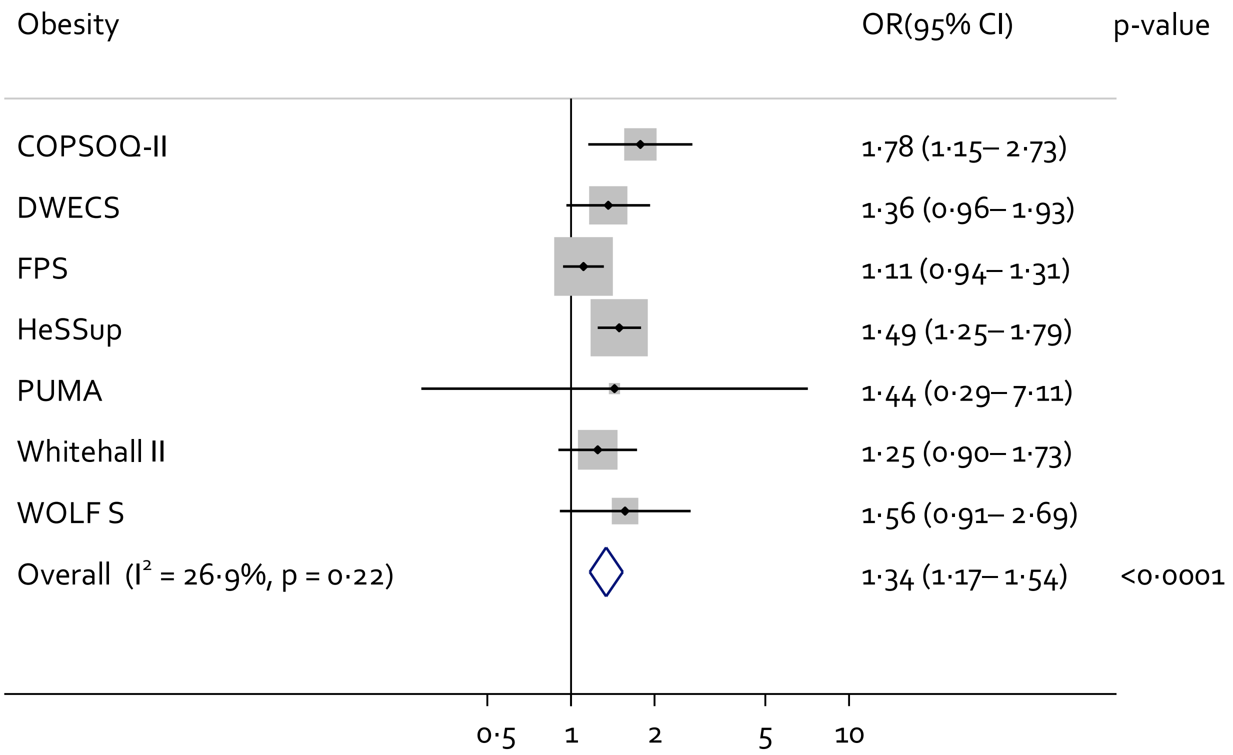

eFigure 3. Age, sex and socioeconomic status adjusted association between long working hours and sedentary lifestyle at baseline

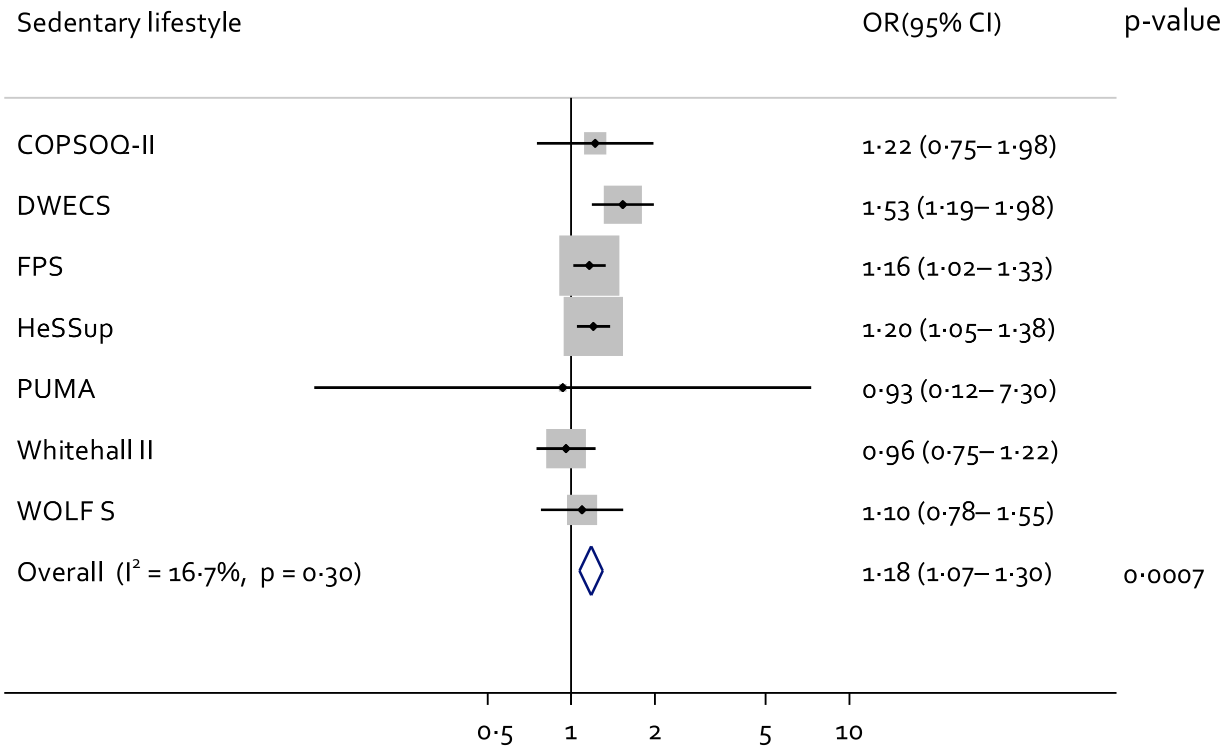

eFigure 4. Age, sex and socioeconomic status adjusted association between long working hours and smoking at baseline

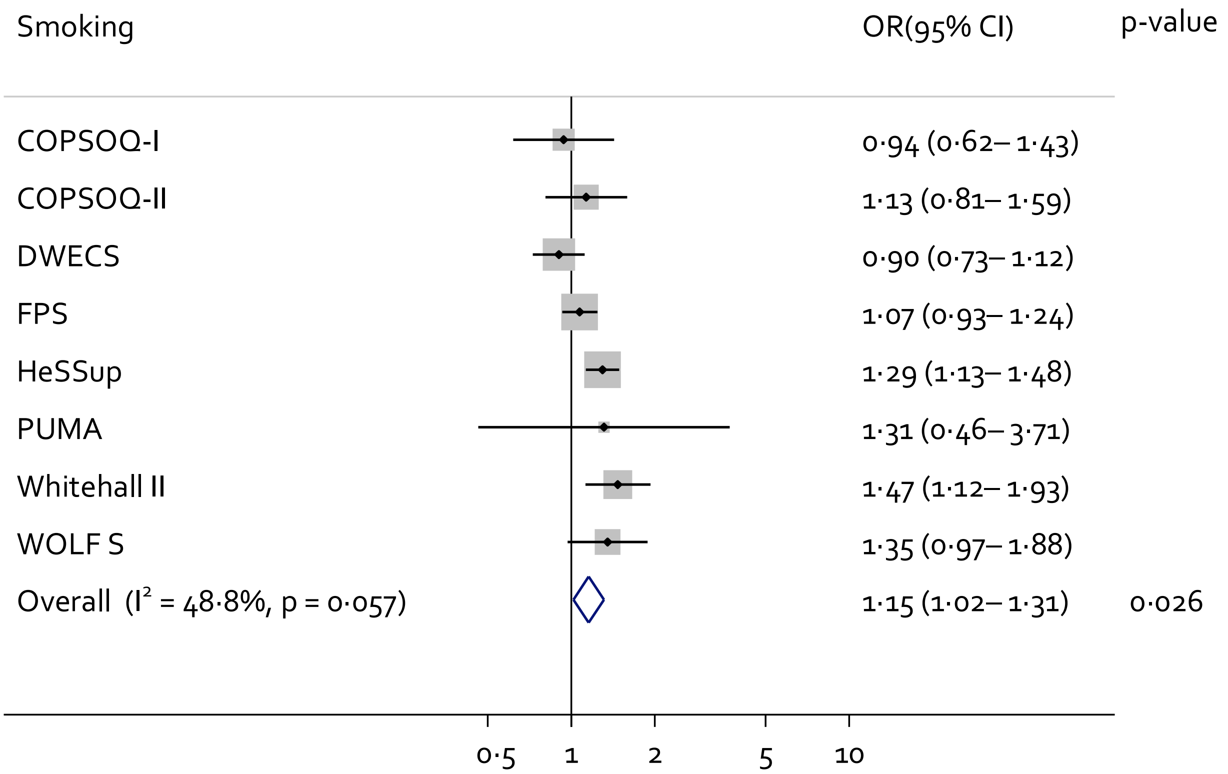

eFigure 5. Age, sex and socioeconomic status adjusted association between long working hours and non-drinking at baseline

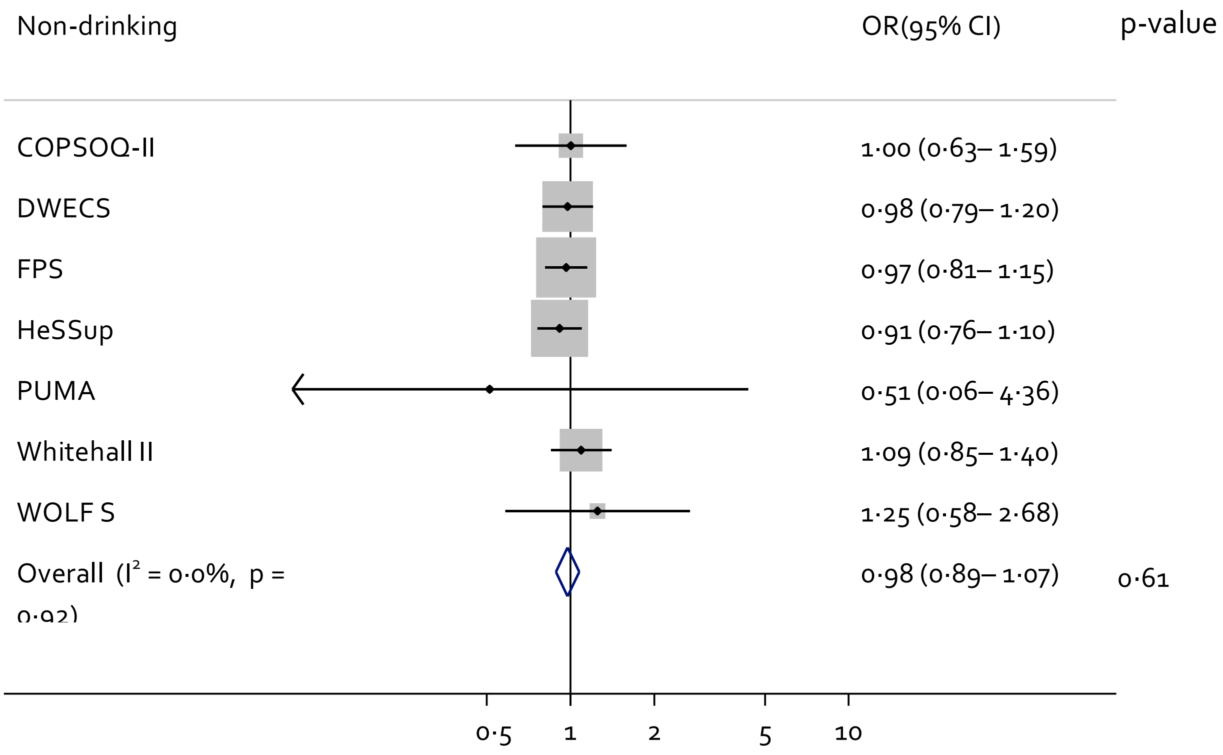

eFigure 6. Age, sex and socioeconomic status adjusted association between long working hours and risky drinking at baseline

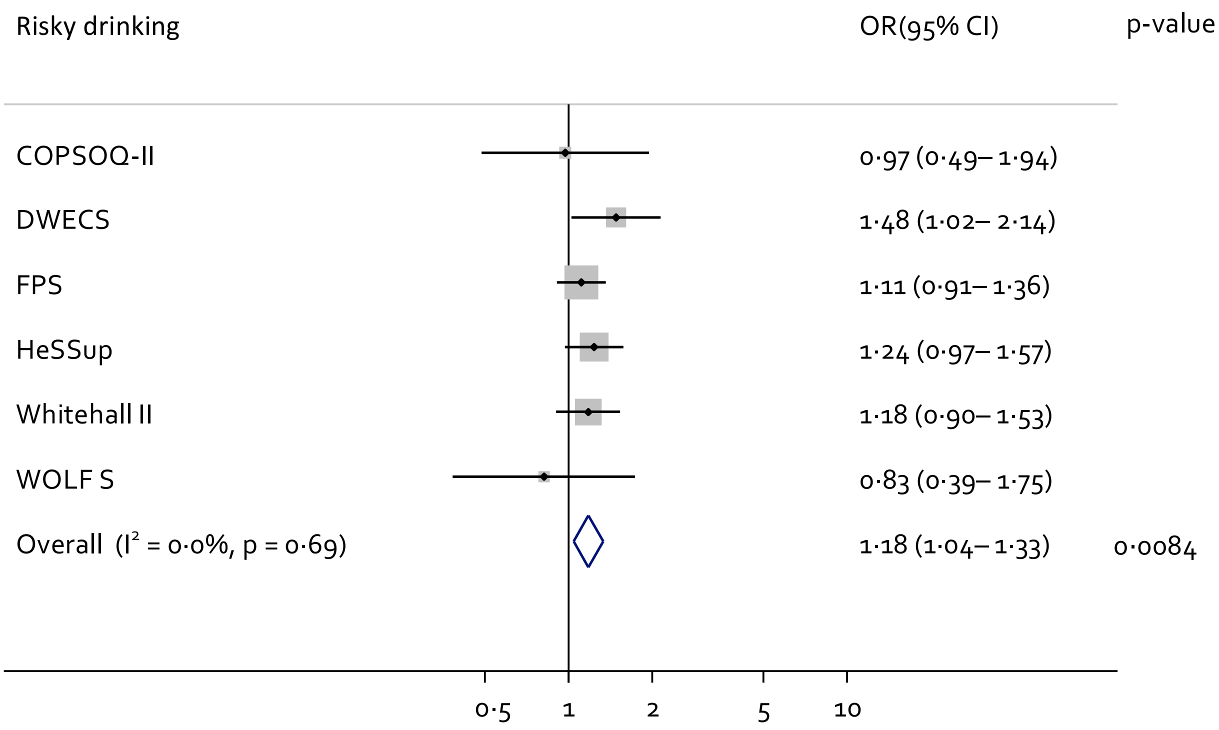

Web appendix 5: Multivariable adjusted random-effects meta-analyses of long working hours with incident atrial fibrillation

eFigure 7. Random-effects meta-analysis of the association of long versus standard working hours with incident atrial fibrillation adjusted for age, sex, socioeconomic status and lifestyle-related factors (COPSOQ I was not included due to missing data on physical activity and alcohol use)

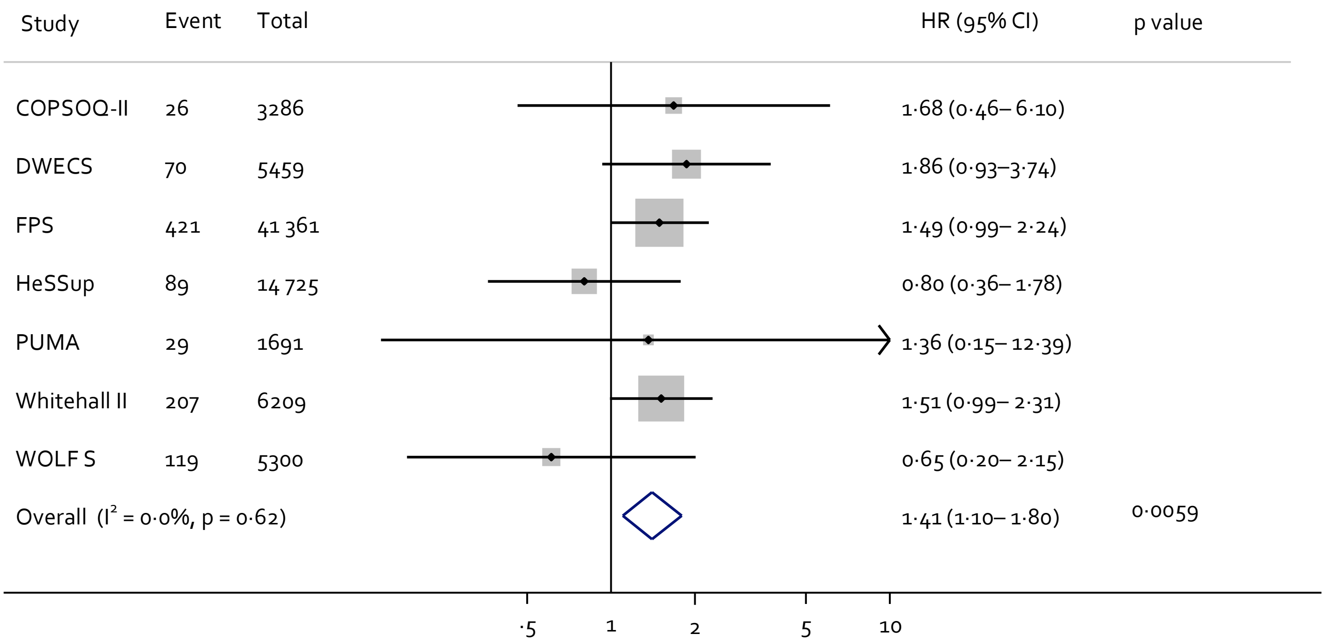

Supplement: Supplementary Appendix [file appendix_ehx324.pdf]
